# Digital Translation Platform (Translatly) to Overcome Communication Barriers in Clinical Care: Pilot Study

**DOI:** 10.2196/63095

**Published:** 2025-03-14

**Authors:** Victor Olsavszky, Mutaz Bazari, Taieb Ben Dai, Ana Olsavszky, Fabian Finkelmeier, Mireen Friedrich-Rust, Stefan Zeuzem, Eva Herrmann, Jan Leipe, Florian Alexander Michael, Hans von Westernhagen, Olivier Ballo

**Affiliations:** 1 Department of Dermatology, Venereology and Allergy University Medical Center and Medical Faculty Mannheim Heidelberg University Mannheim Germany; 2 Translatly UG Offenbach Germany; 3 Clinic for General and Visceral Surgery Vivantes Klinikum Kaulsdorf Berlin Germany; 4 Department of Internal Medicine 2, Hematology/Oncology Goethe University Hospital Frankfurt am Main Germany; 5 Department of Internal Medicine 1, Gastroenterology, Hepatology and Endocrinology Goethe University Hospital Frankfurt am Main Germany; 6 Institute of Biostatistics and Mathematical Modeling Goethe University Hospital Frankfurt am Main Germany; 7 Department of Medicine V, Division of Rheumatology University Medical Center and Medical Faculty Mannheim Heidelberg University Mannheim Germany

**Keywords:** language barriers, health care communication, medical app, real-time translation, medical translation

## Abstract

**Background:**

Language barriers in health care can lead to misdiagnosis, inappropriate treatment, and increased medical errors. Efforts to mitigate these include using interpreters and translation tools, but these measures often fall short, particularly when cultural nuances are overlooked. Consequently, medical professionals may have to rely on their staff or patients’ relatives for interpretation, compromising the quality of care.

**Objective:**

This formative pilot study aims to assess the feasibility of Translatly, a digital translation platform, in clinical practice. Specifically, the study focuses on evaluating (1) how health care professionals overcome language barriers and their acceptance of an on-demand video telephony platform, (2) the feasibility of the platform during medical consultations, and (3) identifying potential challenges for future development.

**Methods:**

The study included ethnographic interviews with health care professionals and an observational pilot to assess the use of the Translatly platform in clinical practice. Translatly was developed to make real-time translation easy and accessible on both Android and iOS devices. The system’s backend architecture uses Java-based services hosted on DigitalOcean. The app securely exchanges data between mobile devices and servers, with user information and call records stored in a MySQL database. An admin panel helps manage the system, and Firebase integration enables fast push notifications to ensure that health care professionals can connect with translators whenever they need to. The platform was piloted in a German university hospital with 170 volunteer nonprofessional translators, mainly medical students, supporting translation in over 20 languages, including Farsi, Dari, and Arabic.

**Results:**

Ethnographic research conducted by interviewing health care professionals in Frankfurt am Main and other German cities revealed that current practices for overcoming language barriers often rely on family members or digital tools such as Google Translate, raising concerns about accuracy and emotional distress. Respondents preferred an on-demand translation service staffed by medically experienced translators, such as medical students, who understand medical terminology and can empathize with patients. The observational pilot study recorded 39 requests for translation services, 16 (41%) of which were successfully completed. The translations covered 6 different languages and were carried out by a team of 10 translators. Most requests came from departments such as infectious diseases (5/16, 31%) and emergency (4/16, 25%). Challenges were identified around translator availability, with 23 (59%) total requests (N=39) going unanswered, which was further evidenced by user feedback.

**Conclusions:**

This pilot study demonstrates the feasibility of the Translatly platform in real-world health care settings. It shows the potential to improve communication and patient outcomes by addressing language barriers. Despite its potential, challenges such as translator availability highlight the need for further development.

## Introduction

Language barriers in health care can have a profound impact on patient management. Patients who are not fluent in the primary language of the health care facility are at higher risk of misdiagnosis due to miscommunication during history taking or description of symptoms [1]. This can lead to inappropriate treatments and interventions, worsening the patient’s condition [2]. In addition, there may be an increased risk of medical errors or even an increased risk of a serious medical event [3]. Patients may feel ignored, excluded, or misunderstood, leading to reduced satisfaction with care. Language barriers can also hinder the development of trust between the patient and the health care provider [4]. Clinic staff, by contrast, complain of frustration due to suboptimal treatment and the additional time spent with patients as a result of miscommunication [5]. To address these challenges, many health care facilities employ interpreters or multilingual staff and use written materials, simplified language, or even online translation tools [6-8]. Difficulties in communication and misunderstandings can result in additional costs [9], ultimately borne by hospitals, statutory health insurance funds, and the health care system as a whole.

In Germany, there are more than 6 million people with limited knowledge of German. More importantly, there are no or only inadequate translation services available in medical facilities. A study commissioned by the Federal Anti-Discrimination Agency summarizes the role of communication barriers as a risk of discrimination in health care, based on data from 2010 to 2020 in Germany [10]. One of the main issues raised is the lack of efficient access to professional translators for German health services. However, given that Germany is currently home to nearly 22 million people with a migrant background, and that an estimated 25% of German citizens speak 2 foreign languages in addition to German [11], this hidden potential of language skills can be reflected in health care professionals such as doctors, medical students, nurses, and medical technicians. At present, this diversity of foreign language skills is either not used at all or only used in exceptional situations that occur by chance to overcome communication obstacles in the medical sector. In addition, the shortage of health care professionals [12] will lead to a further increase in the number of people with foreign language skills in German society due to immigration in the coming years [13].

The problem of language barriers in health care is not only confined to Germany. As the world’s population becomes increasingly diverse and migratory, the need to ensure effective patient-provider communication in a multilingual context is growing. Other countries with a long history of immigration have faced similar challenges in their health care systems. In Canada, for instance, the Winnipeg Regional Health Authority, a local health care organization, employs trained health interpreting staff for in-person and telephone interpreting [14]. While such language services are useful proactive policies, they are not available in all Canadian communities [15] and cannot meet the most common ad hoc interpreting needs [16]. In the United States, state laws require health care facilities to provide interpretation services for patients with limited English proficiency, but these requirements cannot be adequately met [17]. Finally, in Australia, almost half of all clinical practices make use of the translation and interpreting service, which is specifically funded by the government to address language barriers in health care [18]. While these examples provide valuable insights and potential solutions that Germany could consider in its efforts to address its own unique challenges related to language barriers, none of these possible approaches take into account the nuances of dialect or the importance of cultural context in communication. This is where technology-enabled solutions, such as Translatly (Translatly UG), play a critical role in addressing these barriers more effectively. Translatly harnesses the existing, underutilized linguistic potential within the health care workforce, offering an innovative and scalable solution to overcome communication obstacles. With more than €400 (US $436) billion spent on health care in Germany each year [19], digitally targeting these resources to overcome translation barriers could have a positive impact not only on health care delivery, but also on health care expenditure.

With this formative pilot study, we aim to assess the feasibility of Translatly, a digital eHealth platform designed to address language barriers in everyday clinical practice in Germany. Our approach focuses on implementing this digital on-demand translation platform for clinics. The main research questions of the study are as follows: (1) What are the current practices of health care professionals in overcoming language barriers and do they accept an on-demand medical translation platform? (2) Is the Translatly platform feasible for use in clinical settings to overcome language barriers? (3) What other challenges may arise in the use of Translatly that can be addressed in future studies? Translatly is a mobile and tablet app developed in Germany by a founding team of doctors, technicians, and entrepreneurs to facilitate live translation in everyday medical practice by virtually connecting qualified language mediators with doctors and their patients. This platform allows in-house health care professionals with foreign language skills to register as translators who can be requested and engaged via video telephony when communication barriers arise during doctor-patient interactions. By providing an accessible, scalable, and efficient solution for translation, Translatly has the potential to not only improve the care and management of nonnative patients but also reduce health care costs, making it a sustainable solution for the future.

## Methods

### Study Design

This formative pilot study used a mixed methods approach combining qualitative ethnographic interviews and observational data collection methods to assess the feasibility of the digital translation platform Translatly in a clinical setting. To explore the extent of language barriers in health care and how they are managed in daily practice, an extensive opinion survey was conducted using an ethnographic questionnaire at the University Hospital of the Goethe University in Frankfurt am Main, as well as in private practices in the Frankfurt area and other selected cities.

The findings from these interviews were used in the design and development of the platform. An observational pilot study was then conducted to test the feasibility of the platform in real medical settings. The use of the Translatly platform was monitored and recorded without manipulating any variables, focusing on how it was integrated into the routine clinical management of patients by health care providers. The pilot study took place over 2 months, from December 1, 2022, to January 31, 2023, at the University Hospital of the Goethe University in Frankfurt am Main.

### Study Participants

Participants were recruited using convenience sampling. Health care professionals, including doctors, physiotherapists, and psychotherapists, participated in interviews using an ethnographic research questionnaire. They were identified and contacted through 2 primary methods: first, practitioners at the University Hospital of the Goethe University in Frankfurt am Main were approached directly; second, health care professionals from private practices in the Frankfurt and Offenbach areas were contacted by email, followed by telephone calls to those who expressed interest. Contact details for these private practices were obtained from public directories maintained by the University Hospital of Goethe University. Additionally, practitioners in Düsseldorf and the United States were contacted through recommendations from respondents.

Locating volunteer translators within the health care workforce and matching them with medical staff presented a technical challenge. To ensure the platform’s optimal function, a substantial pool of volunteer translators was needed. Recruitment focused on medical students, as they possess the medical knowledge to handle complex translations [20]. They were enrolled through a clinical elective course titled “Translatly and Digitalization in Healthcare,” which also provided them with community service certification. Volunteer translators were briefed on data protection regulations, including General Data Protection Regulation (GDPR) compliance.

By the last quarter of 2022, a total of 170 volunteer translators had been registered, 90% (n=153) of whom were medical students from the University Hospital. The remaining 10% (n=17) comprised medical staff. For the pilot study, translators were recruited for the following languages: Farsi, Dari, Arabic, Italian, Urdu, Portuguese, Spanish, Turkish, Russian, Albanian, Vietnamese, Romanian, Slovenian, Pashto, Macedonian, French, Croatian, Hindi, Bosnian, Azerbaijani, Korean, and Berber.

Patients were recruited through direct contact with doctors at the University Hospital, who identified cases needing translation services. Doctors interested in participating were provided instructions on how to use the Translatly app, and patients were also supported in using the app if needed.

### Data Collection

#### Overview

Data were collected through ethnographic interviews with health care professionals using a questionnaire, as well as observational monitoring of the Translatly platform in clinical practice during the pilot study. The interviews focused on current practices for overcoming language barriers, while the observational data recorded translation requests, languages, and involved medical departments.

The ethnographic questions were designed specifically for this study and followed the format of semistructured interviews (Multimedia Appendix 1). This approach provided a mix of open-ended questions and specific information requests, allowing for both structured inquiry and the flexibility for interviewees to express their own experiences and opinions freely. Specifically, practitioners were asked about their personal experiences, their challenges in dealing with non–German-speaking patients, and their thoughts on how to improve communication. Questions about the use of software and technology, and specific instances where translation services have been required, provide deeper insights into everyday practice and the issues involved. Typical of ethnographic interviews, the questionnaire can be used in a context where interviews are more informal and conversational. The interviews were designed to last approximately 50 minutes, with time allocated for each section of the interview guide.

The pilot study collected data on the frequency of requests for translation services, the response rate to these requests, the duration and languages of completed translations, and the hospital departments making these requests. User feedback was also collected to assess problems and challenges during the pilot phase. This information allows for a comprehensive assessment of the use of the Translatly platform in clinical practice by providing data on the demand for translation services, the availability of the translator network, and the different medical specialties requiring translations. As this feasibility study only aims to measure and observe whether Translatly can be used as a translation platform in everyday clinical practice, a comparison group (eg, patients without a translation platform) was not required.

#### Instruments

Data from ethnographic interviews were collected through written notes and supplemented by audio recordings to ensure accuracy and completeness. The interviews were then transcribed into words for analysis.

The tools required for the observational pilot study were the Translatly app and a mobile device or tablet. While some medical departments at the University Hospital of the Goethe University in Frankfurt am Main provided their own tablets, this was not a requirement as the app could be used by doctors, patients, and translators on their own smartphones. Translatly was preinstalled on all devices used in the pilot study with the help of the study team. Network connectivity was provided by the university hospital’s wireless network or by the mobile phone’s network provider.

### Analytical Techniques and Software

#### Qualitative Content Analysis

A qualitative content analysis was conducted to systematically evaluate the data from the ethnographic interviews. This analysis provided insights into clinical standards, patient documentation, and the use of translation services.

The analysis of the pilot study data was performed using Excel (version 2408 build 16.0.17928.20114, 64-bit; Microsoft Corp.), with raw data provided by the Translatly digital platform, which is methodologically described in detail below.

#### Operating Mode of the Translatly Digital Translation Platform

**Figure 1:**
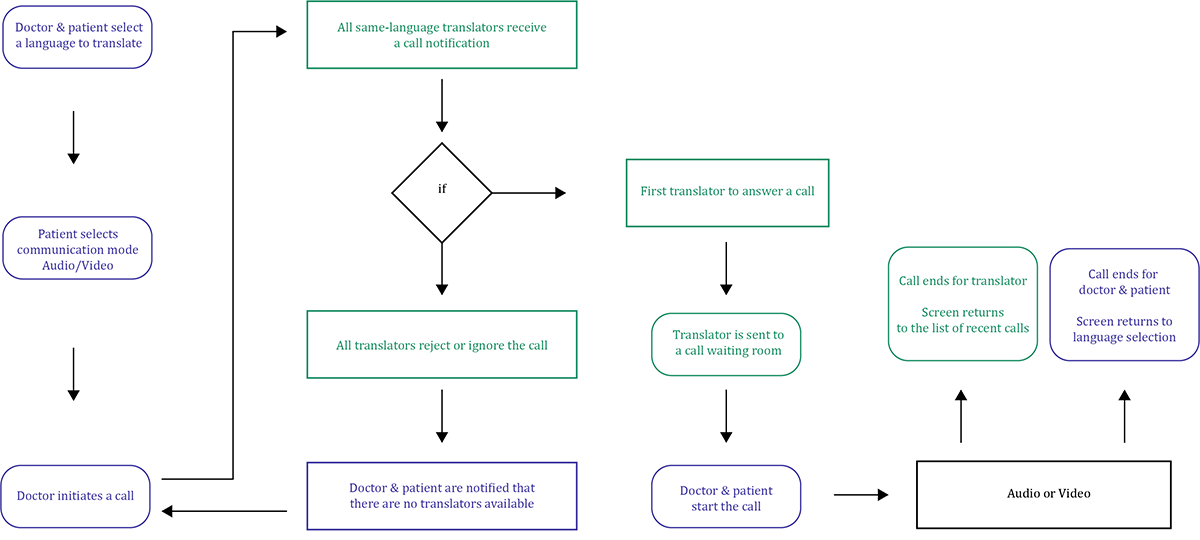
Flowchart describing the operational process of the Translatly digital translation platform. It starts with a medical practitioner (doctor) identifying the need for a translator during a patient consultation and selecting the desired language for translation using the Translatly app on a smartphone or tablet. Once the patient selects their preferred mode of communication, either audio or video, the Translatly algorithm is activated and searches its database for available translators who have registered as proficient in the required language. The algorithm sends a contact request to these translators. The translators receive the request on their devices and the first one to accept the request is connected to the doctor and the patient. If the translator is accepted, an audio or video call is initiated. If all the translators reject the request or do not respond within a certain time frame, the algorithm notifies the doctor and the patient that the search was unsuccessful. At the end of the call, the translator's screen returns to the list of recent calls and the doctor's and patient's screen returns to language selection. Doctor and patient process operations are shown in purple, while translator operations are shown in green.

Translatly’s digital translation platform is designed to provide medical facilities, such as hospitals or private practices, with an app that can be used on smartphones or tablets within the facility. With the app, doctors or other medical staff treating patients can access a pool of translators who can provide language assistance during patient consultations. The medical practitioner chooses the language to be translated and the Translatly algorithm selects and contacts the appropriate translators from the Translatly pool (Figure 1). The first translator to accept the contact request is connected directly to the treating team. The medical staff, patient, and translator communicate via Translatly’s internal audio or video interface.

#### Translatly’s Digital Platform Development Framework

The initial steps to ensure the successful implementation of the digital platform before the full implementation of the pilot phase consisted of 3 main pillars: (1) evaluation of the feasibility of a digital interaction-translation platform in a German university hospital setting; (2) regulatory compliance research; and (3) app design and development.

Following an extensive review of the existing medical literature, the feasibility assessment (pillar 1) consisted of developing the ethnographic research questionnaire (Multimedia Appendix 1) to determine the impact of language barriers in health care and how they are managed in daily practice.

Regulatory compliance research (pillar 2) involved an in-depth assessment of relevant medical regulations and standards in Germany. In brief, our team ensured that the app complies with regulatory requirements for data security and privacy. In addition, we ensured that our research project was in line with the purpose of the Innovation Fund, which was established within the German Federal Joint Committee to promote new forms of health care that go beyond the existing standard of care covered by statutory health insurance in Germany [21].

Design thinking workshops, cofacilitated by members of the clinical research team and software engineering specialists, marked the start of the design and development phase (pillar 3). These sessions set the stage for a subsequent design sprint phase. During this phase, the software development team built and evaluated prototypes and integrated and validated the selected ideas for the app. The research team consisted of a multidisciplinary collective, including 2 software developers with computer science expertise, 2 clinical oncologists to provide medical insight, a clinical trial investigator to oversee research integrity, and a business engineer to manage regulatory issues.

#### Translatly Service System Architecture

**Figure 2:**
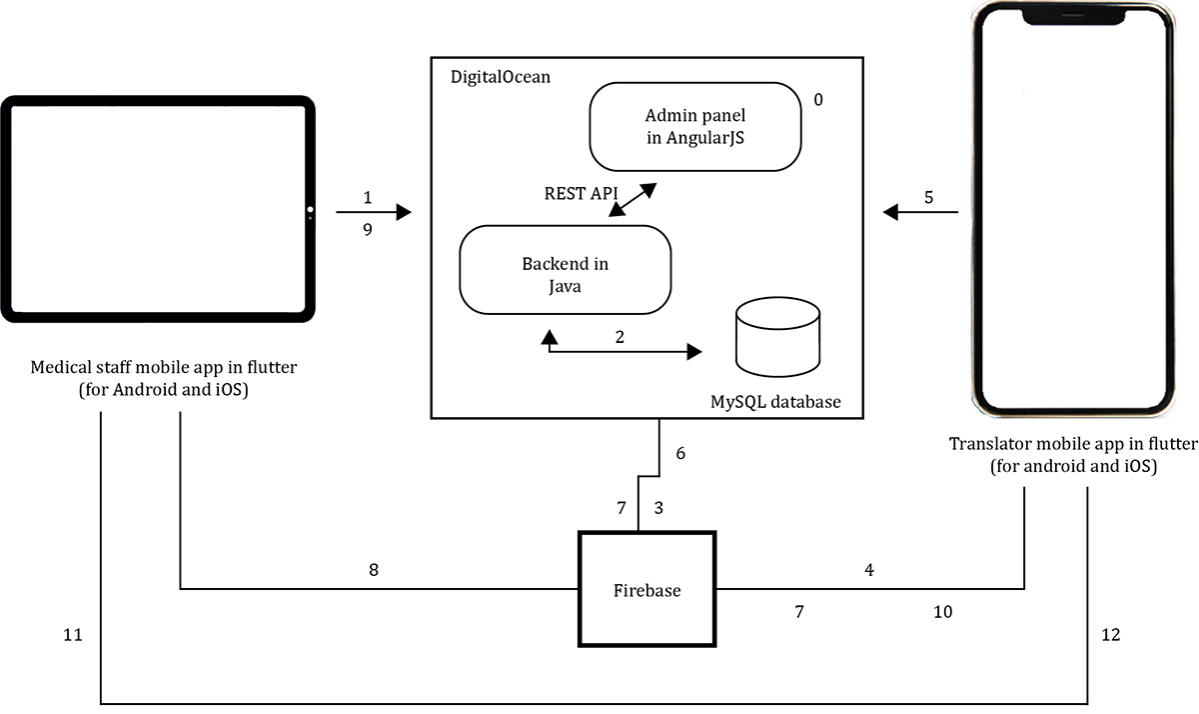
Architecture and data flow of the Translatly Digital Interaction Platform. This diagram illustrates the systematic process from the initiation of a translation request to the establishment of a video telephony session between medical staff and translators. Each step (0-12) marks a critical interaction between components of the Translatly platform, ensuring an efficient and fast response to overcome language barriers in real-time health care scenarios. “0. Administration panel”: Initially developed with AngularJS for the front end, Java Spring for the backend, and integrated with a MySQL database, the administration panel is used by administrators to create, edit, and delete accounts for medical staff and translators. “1. Language request”: The operator uses the mobile app to send a Representational State Transfer (REST) request for translation services to the backend hosted on DigitalOcean. ”2. Translator list retrieval”: Upon receiving the request, the backend accesses the MySQL database to retrieve a list of available translators who speak the required language. “3. Translator notification request”: The backend sends a request to Firebase, the cloud service, to initiate a push notification to the appropriate translators. “4. Push notification to translators”: Firebase processes the request and sends a push notification to the translators' mobile devices. “5. Translator availability confirmation”: The first translator to respond sends an availability confirmation back to the backend via the REST application programming interface (API). “6. Notification of translator confirmation”: Upon confirmation, the backend updates the Firebase cloud service with the translator's availability. “7. Cancellation notification to translators”: Firebase will then send a push notification to the remaining translators to cancel the call as a translator has been found. “8. Notification of attending staff”: Firebase also notifies the medical staff that a translator has been secured for the session. “9. Meeting link to medical staff”: The medical staff member receives the meeting link from the backend after joining the call. “10. Meeting link to the translator”: Similarly, the translator receives the meeting link via a push notification from Firebase. “11. Medical staff meeting join”: The medical staff member joins the video chat meeting using the link provided. “12. Translator meeting join”: The translator also joins the video chat meeting so that the translation session can begin.

To ensure the desired functionality of the Translatly digital interaction platform, a high-level software, database, and server architecture have been defined and implemented by our software development team (Figure 2). To achieve cross-platform compatibility on both Android (Google LLC/Alphabet Inc.) and iOS (Apple Inc.) devices, the architecture consists of 2 main mobile apps developed using the Flutter framework [22]. One app is designed specifically for medical staff, acting as a practitioner interface, while the other acts as a translator interface, supporting real-time communication via audio or video telephony.

The platform’s backend is built on Java-based services, hosted on DigitalOcean (DigitalOcean Holdings, Inc.) for a stable and scalable cloud infrastructure [23]. The services can be accessed through a REST API (Representational State Transfer Application Programming Interface) [24] protected by an OAuth (Open Authorization) algorithm [25] that secures the connection between the mobile apps and the server, ensuring efficient data transfer and real-time interactions. Moreover, a comprehensive administrative panel, originally developed with AngularJS (Angular Java Script) [26] and later migrated to ReactJS (React Java Script) [27], provides full system management and is responsible for maintaining user profiles, language preferences, and service availability of registered translators.

For data persistence, a MySQL (Oracle Corporation) database is used to store structured data, handling user information, interaction records, and call history [28]. To facilitate push notifications, we have integrated Firebase (Google LLC/Alphabet Inc.) into our system architecture [29]. This integration enables in-house health care professionals to be promptly notified and instantly connected to ongoing consultations. Real-time database and user authentication are implemented internally. Finally, Twilio (Twilio Inc.) is used as the video communication tool, known for enabling developers to build custom communication solutions [30].

#### Translatly User Experience Design and Interface Functionality

To ensure the intended mode of operation, we have opted for an intuitive user interface, specifically designed to guarantee and immediately establish an optimal interaction between medical professionals and translators. Upon opening the app, medical professionals must enter their credentials provided by our team to securely access Translatly’s services (Multimedia Appendix 2). After logging in, they are presented with the main app screen, which prominently features an on-demand translation tab with an extensive language selection menu. Next, the medical staff user can choose between audio and video call modes, conceivably in the presence of and in agreement with the patient. The interface then displays the status of the translator call process and notifies the medical staff when a translator has accepted the call, providing an easy transition into the call.

**Figure 3:**
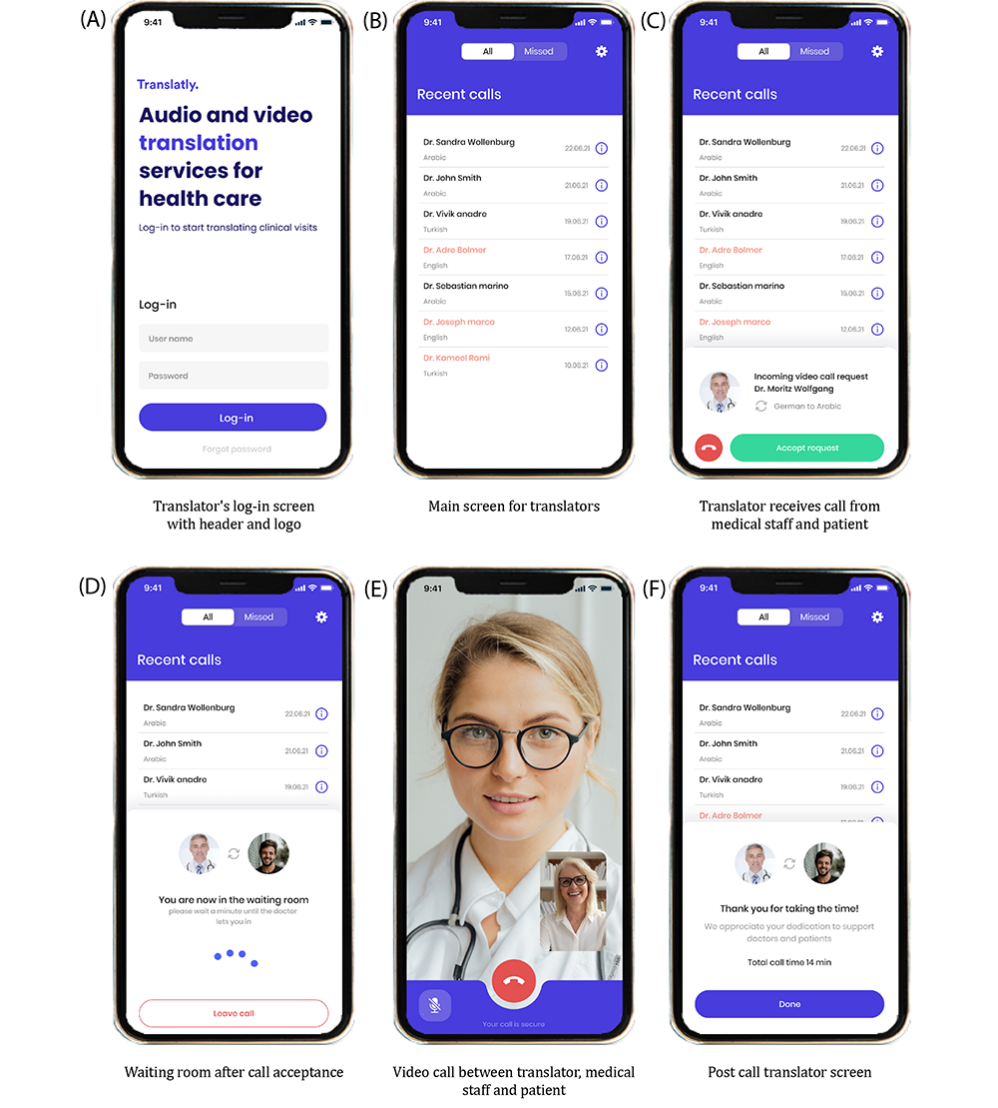
Selection of Translatly mobile frontend screenshots showing different stages of the user experience for translators. (A) Log-in screen with header and logo, where translators enter their credentials to access the platform. (B) The main screen displays the last calls and missed calls of the translator, including the language that was interpreted during the call and the name of the doctor. (C) Notification when a translator receives a video call request from a health care professional, indicating the languages to be translated. (D) Upon acceptance of the call, the translator is directed to a waiting room and is awaiting entry into the consultation. (E) Active video call interface used by the translator to communicate with medical staff and patient. (F) The postcall screen provides a summary of the call duration and a thank you message with an option to end the session.

On the other side, translators access their dedicated portal through a similar secure log-in interface (Figure 3A). Their main screen displays recent and missed calls, with relevant details such as the caller’s name and the languages translated during the respective calls (Figure 3B). An incoming call is displayed as a slide-up in-app notification with relevant information (Figure 3C). Upon acceptance, the translator is temporarily held in a virtual waiting room until the calling user agrees to start the call (Figure 3D). After the call (Figure 3E), a postcall notification provides a summary of the call (Figure 3F).

### Ethical Considerations

This study involving human participants has been reviewed and approved by the Medical Ethics Committee of the Goethe University, Frankfurt am Main, Germany (ethics approval 2024-1776). The study was conducted in accordance with the principles of the Declaration of Helsinki. All participants provided informed consent for their involvement in the study. Additionally, the Medical Ethics Committee has approved the secondary analysis of the pilot phase data without the need for further consent. We confirm that the Translatly team adheres to regulations, including compliance with the GDPR, the Federal Data Protection Act, the Hessian Data Protection Act, and confidentiality in accordance with § 203 of the German Criminal Code. In addition, all data collected are anonymized. No compensation was provided to participants in this study. All images used in this study are open source and publicly available on the official Translatly website [31], with no identifiable individual participants or users depicted.

## Results

### Summary of Responses to Ethnographic Research Questionnaires

The ethnographic research was conducted to assess the impact of language barriers in health care, and to gain a holistic view of the challenges health care professionals face in their clinical routine when dealing with nonnative speaking patients. Respondents included specialists and therapists from maximum care hospitals or university hospitals (6/10, 60%), from private practice (3/10, 30%), or from both the private and public sectors (1/10, 10%; Multimedia Appendix 3). The majority of respondents were oncologists (4/10, 40%) from major German cities such as Offenbach am Main (2/10, 20%), Frankfurt am Main (1/10, 10%), Düsseldorf (1/10, 10%), and even from the United States (1/10, 10%). We were able to identify a number of key findings from the health care professionals we interviewed, which were later of great importance for the design and development of the app. Most respondents used both medical staff and family members (5/10, 50%), but no other formal medical translation services such as third parties or software (9/10, 90%), when facing language barriers in their workplace (Multimedia Appendix 3). Common foreign languages encountered in medical settings in Frankfurt am Main and other selected German cities were Russian, Kurdish, Arabic, French, Turkish, Afghan, Italian, Portuguese, Eritrean, Serbian, Croatian, Bulgarian, Spanish, Greek, and Chinese.

Most respondents raised concerns about accuracy and the emotional burden on relatives, with some giving specific examples of relatives hiding the truth from patients to appease them. Some professionals used digital tools such as Google Translate (Google LLC/Alphabet Inc.), but with dissatisfaction due to the lack of confidence in the accuracy of the translation and the lack of empathy associated with using a device, particularly when making difficult diagnoses. The availability of hospital interpreter pools in larger hospitals was noted but underutilized, mostly due to complex technicalities. Notably, some doctors working in private practice stated that, in the case of non–German-speaking patients, they would offer the patient a new appointment and ask them to bring an interpreter with them.

When considering either a scheduled appointment or an on-demand call, health care professionals expressed a preference for an on-demand translation service such as Translatly (8/10, 80%; Multimedia Appendix 3). The ideal translation solution was identified as a pool of medically experienced translators, suggesting that medical staff, including medical students, would be most effective due to their knowledge of medical terminology and empathy. Estimated usage of a translation app varied, with most professionals predicting usage of at least once a week. Finally, concerns about bureaucratic and logistical hurdles, the efficiency of nonmedical translators, and the overall feasibility of integrating such a system into daily medical practice were raised, despite the enthusiasm for a digital translation platform.

### Evaluation of Pilot Study

The pilot study recorded 39 translation requests over a 2-month period, of which 16 (41%) were successfully completed (Table 1).

The successfully completed translations accounted for a cumulative duration of 209 minutes across 16 distinct conversations. These translation sessions were carried out by a team of 10 translators, who contributed to facilitating communication across language barriers within the hospital setting. The breakdown of languages translated through the platform included Russian, Ukrainian, Serbian/Croatian/Bosnian, Arabic, Greek, and Urdu, reflecting a wide range of linguistic needs among the patient population.

The origin of translation requests spanned several medical departments, with the Department of Infectious Diseases submitting the highest number of requests (n=5), followed by the Emergency Department with 4 requests. Internal medicine departments submitted 3 requests, while pediatrics and psychiatry departments each submitted 2 requests for translation services.

**Table 1:** Overview of translation requests during the pilot phase of the implementation of the digital translation platform Translatly at the Goethe University Hospital in Frankfurt am Main (December 2022-January 2023). The table summarizes the total number of on-demand translation requests, the outcome of these requests (whether completed or unanswered), the languages translated, and the medical departments from which the requests originated. The study involved health care professionals from different departments and focused on the feasibility of the Translatly platform in a clinical setting.

Description			Values (n=39), n (%)
**Translation requests**			
	Remained unanswered	23 (59)	
	Successfully completed	16 (41)	
	Started, but not completed	0 (0)	
**Translated languages (n=16)**			
	Russian	6 (38)	
	Ukrainian	3 (19)	
	Serbian/Croatian/Bosnian	2 (13)	
	Arabic	2 (13)	
	Greek	2 (13)	
	Urdu	1 (6)	
**Medical departments of translation requests (n=16)**			
	Infectious diseases	5 (31)	
	Emergency department	4 (25)	
	Internal medicine departments	3 (19)	
	Pediatrics	2 (13)	
	Psychiatry	2 (13)	

### Challenges of Using the Translatly Platform

Several challenges were identified during the pilot study. A key issue was translator availability, with 23 out of 39 (59%) translation requests going unanswered, mainly due to translators being unavailable at the time of the request. In addition, while feedback from both medical staff and translators was generally positive—particularly regarding the simple and intuitive design of the platform—some users expressed concern about the delayed responses. Notably, some translators indicated that they had been contacted via instant messaging beforehand, to ensure availability when a translation request was made.

Taken together, the results of this study addressed the 3 primary research questions: (1) how health care professionals overcome language barriers and their acceptance of Translatly’s on-demand medical translation platform, (2) the feasibility of implementing Translatly in clinical settings to overcome language barriers, and (3) the identification of potential challenges that could inform future studies.

## Discussion

### Principal Findings

With this formative research study, we present the first successful implementation of the digital translation platform “Translatly” in a medical setting to tackle language barriers. Ethnographic research revealed that health care professionals currently rely on family members or online tools to overcome language barriers. There was a strong preference among health care professionals for an on-demand translation service staffed by medically experienced professionals with foreign language skills. The pilot study, conducted at the Goethe University Hospital in Frankfurt am Main, Germany, demonstrated the feasibility of using Translatly in a real-world clinical setting. Translatly’s solution is to virtually enable patient-doctor communication by leveraging the language skills of health care professionals. Facilitating real-time translation services during patient consultations directly addresses the problems of miscommunication in health care by providing immediate access to translators, who in this study are either medical students or trained medical professionals. Our translator pool, which is predominantly made up of migrant health professionals, not only ensures that complex medical terminology is translated properly, but can also address cultural language differences [32], thereby reducing the likelihood of misunderstandings and subsequent medical errors.

To the best of our knowledge, our digital approach to addressing the problems of miscommunication due to language barriers in health care is unique in that it seeks to combine human language skills with the capabilities of modern eHealth technology. Currently, approaches to the problem of language barriers focus heavily on so-called machine translation (MT) software, which is specifically designed to translate text or speech from one language to another without human intervention [33,34]. In fact, medical translation apps have been published or are already available in app stores. However, they either rely on MT or contain predefined health phrases [35]. The modern focus on automated translation alternatives is mainly motivated by the fact that human translators can be costly and time-consuming [36]. This is evidenced by studies that have shown, for example, that the use of human interpreters has decreased in several health centers in the United States from 2009 to 2019 [37]. Therefore, the Translatly platform represents a novel solution that does not require translators to be tied to a specific hospital, and moreover, the intensive search for a specific translator can be quickly overcome by accessing the translator pool, which can be done by medical staff directly from the patient’s side of the bed.

One of the key findings from the responses of the medical staff interviewed using our ethnographic research questionnaires was that the ideal solution proposed by the majority of interviewees would be to actually use medical staff or medical students with foreign language skills, and this was a key influence in prompting our team to look for volunteer translators to join our translator pool. However, we acknowledge that this could be a major limitation of our pilot study as the volunteer translators we recruited are not specialist interpreters but rather medical practitioners or students. Therefore, the issue of variability in the medical knowledge and language skills of the translators recruited for this study is a critical concern that could affect the quality and accuracy of translations in clinical settings. Regarding the foreign language skills of volunteer translators with a migrant background, it has been estimated that in 2023 in Germany, approximately 64.5% of people with a migrant background were first-generation migrants, and therefore native speakers of their own language [38]. Native speakers do not normally need language certificates, as they are assumed to have an innate and comprehensive understanding. A native speaker is defined as an individual who typically acquires the language as a young child and maintains it into adulthood [39]. Notably, there are linguists who distinguish between the basic and extended proficiency of native speakers [40]. With regard to the German language skills of translators from migrant backgrounds, there are data showing that in Germany 66% of migrants consider themselves to have a good or professional level of German [41] and strong language skills are required for enrollment in medical faculties. Although these statistics do not resolve the issue of variability in the medical translation skills of the recruited translators, they do support the fact that translation errors via the Translatly platform may indeed be minimal. Contrary to our findings from the ethnographic research questionnaires, other similar interviews showed that hospital staff were strongly opposed to the use of bilingual medical staff services [42]. It should be noted, however, that these interviews were conducted to assess language services for written materials and not for direct dialogue translation. By contrast, we strongly believe that while the Translatly concept does not provide an overarching solution, the use of bilingual medical staff for the Translatly platform represents a significant improvement over the already precarious situation in daily clinical routine, as evidenced by our questionnaire results and others [43], of relying on family members or untrained interpreters. Furthermore, even when official translators are present, there is still potential for mistranslation [44], making other translation alternatives necessary in some cases to improve communication with patients.

To ensure that the Translatly digital platform is user-centric, the needs of the medical staff that emerged from the questionnaires were carefully considered during the design of the operating mode and later during the development of the app. However, as the call is always initiated by the doctor in the presence of the patient, it may be unannounced and unexpected for the translator. We have therefore taken into account the possibility of some translators being unavailable during ad hoc calls and have specifically designed the operating mode and system architecture so that all translators in the pool will receive the calls if they are listed for the selected language to be interpreted. The integration of the Translatly app with Firebase [45], a widely used service for sending push notifications to a large number of users quickly and reliably, helps to facilitate the calling of all required translators from the pool. Moreover, real-time video telephony in clinical settings can be disrupted by connectivity problems due to network instability. Solutions to this issue could include switching to an audio call, which requires less data and can tolerate latency, or reconnecting with another translator via push notifications provided by the robust backend architecture with Firebase. However, these measures may not fully prevent disruptions, so offline text-based translation or rescheduling during outages would be helpful. Further time-saving is achieved through Translatly’s simple, user-friendly app design. Knowing that effective and prompt communication is paramount in the medical setting, we focused on designing our app for the pilot study with simple accessibility, such as few visual elements and features, intuitive navigation, legible fonts, and a few color palettes. As our pilot study aimed to test Translatly’s usability in a real-world medical setting, we had not yet included other app features such as scheduling, an integrated calendar, or chat services.

The results of the pilot study demonstrated a significant need for effective translation services in the medical setting, as evidenced by the Translatly app being used for a total of almost 3.5 hours. The data showed that over 40% (16/39) of translation requests were successfully fulfilled, highlighting the potential of the app to improve communication between health care providers and non–German-speaking patients. Another notable limitation observed during the pilot study was that 23 out of 39 (59%) translation requests went unanswered. This highlights the challenges associated with translator availability and responsiveness, particularly at peak times, which could affect the scalability of such a digital solution across larger or more diverse linguistic landscapes. As a consequence, our pilot study led us to add an optional scheduling feature and calendar to the app. To further mitigate availability issues, we are exploring the possibility of allowing translators to indicate their availability in advance. This system would prioritize periods of high demand and ensure that enough translators are on call when they are most needed. Another option would be a real-time monitoring system that tracks translator availability and dynamically adjusts call routing to match supply with demand. This would help to minimize delays and ensure that translation services are readily available when they are needed. Finally, expanding the pool of translators to include the official translators employed by hospitals and incorporating a translator selection algorithm to improve response rates or using MT-based translation options would ensure broader and more professional coverage. Nonetheless, the diversity of languages requested, from Russian to Urdu, across a range of departments from infectious diseases to psychiatry, underlines the widespread need for effective translation services in medical settings and the successful initial broad implementation of Translatly in a variety of medical departments for patients from diverse linguistic backgrounds.

While Translatly offers many benefits, there are further inherent challenges and limitations that need to be considered. First, to address the issue of variability in the professional translation of medical conversations, more rigorous selection criteria and training protocols for translators are needed. These protocols should include targeted medical terminology training focusing on the most common conditions and procedures in the hospital departments where translation services are critical. In addition, the inclusion of role-playing scenarios with simulated doctor-patient interactions could be implemented to allow students to practice translation in a controlled environment and receive feedback on real-life challenges. Further cultural competency training would also be beneficial, with modules designed to deepen understanding of cultural nuances that may impact on communication, ensuring that translations are both linguistically accurate and culturally sensitive. Next, relying on health care professionals to perform dual roles as medical providers and translators could potentially lead to work overload and burnout. Effective workload management is essential to prevent fatigue and ensure the sustainability of translation services [46]. Therefore, we will consider assessing workload reduction in future studies on the implementation of Translatly.

Collecting robust data on user usage and satisfaction, as well as the impact on treatment outcomes will provide insights that could guide future improvements and adaptations to the platform. In addition, expanding the range of languages offered and scaling the solution to other regions and health care settings could increase its impact, making inclusive health care more accessible nationally and potentially globally. Next, Translatly’s mode of operation does not address the issue of face-to-face interpreting by existing hospital interpreters. While official hospital interpreters can easily be added to Translatly’s pool of translators, we continue to advocate for video interpreting, as recently a systemic review has shown that patient satisfaction with video interpreting is the same as with in-person communication [47].

### Conclusions

This formative pilot study assessed the feasibility of overcoming language barriers in clinical settings through the use of the Translatly digital translation platform. The study aimed to determine how health care professionals overcome language barriers, whether they prefer and accept this on-demand translation solution, and whether it can be effectively integrated into routine clinical workflows. The platform leverages the language skills of medical students and health care professionals, enabling them to serve as on-demand volunteer translators during medical consultations via video telephony. The results showed that Translatly has significant potential to improve communication between health care providers and nonnative speaking patients, thereby improving patient outcomes and optimizing health care resources. Although this early-stage study focused primarily on feasibility, it also identified key challenges, such as translator availability, that can inform future refinements to the platform. The study did not evaluate the quality of translations, nor did it focus on the platform’s architecture or mechanisms for attracting users. Rather, it laid the groundwork for future work by exploring the operational feasibility of Translatly in a real-world health care setting.

Translatly’s alignment with statutory health insurance mandates in Germany positions it as a potential eHealth solution for widespread adoption [48]. By reducing language barriers, it can support better health outcomes and promote health education, particularly in diverse patient populations. A subscription-based model could address the platform’s cost implications, allowing health care facilities to scale the service according to their needs. Initial costs related to technology infrastructure, training, and ongoing maintenance would be manageable, particularly in larger hospitals with robust IT systems, and would be justified by the long-term benefits of the platform, including improved workflow, improved patient outcomes, and reduced overall health care costs. Projected revenue growth of €16,852 (US $18,334) in the first year of implementation, rising to €5,933,455 (US $6,455,302) in the fourth year, supported by strategic expansion and scaling efforts, further underscores the platform’s potential for sustainable financial viability within the health care sector. With further development and strategic implementation, platforms such as Translatly could become an integral part of health care systems, improving communication, patient satisfaction, and reducing health care costs.

Ultimately, Translatly has the potential to set a new standard for overcoming language barriers in health care. By enabling health care professionals to communicate effectively with nonnative speakers, the platform strengthens trust between patients and providers, reduces the emotional burden on families, and supports the delivery of high-quality, culturally competent care.

## Appendix

Multimedia Appendix 1 Ethnographic research questionnaire used for the feasibility study, translated into English.

Multimedia Appendix 2 Examples of screenshots of the Translatly app user interface for medical professionals on a tablet device. (A) The log-in screen with header and logo where medical staff enter their credentials. (B) The main app screen for medical staff, showing an on-demand translation tab with a drop-down menu for language selection. (C) After selecting the language to be translated, the medical staff user can choose between audio or video call mode to initiate a translation session. (D) Screen showing the process of calling available translators once a translation request has been made. (E) Notification screen informing medical staff when a translator has accepted the call, with options to continue or start again. (F) An illustrative example of videoconferencing with a translator during medical consultation.

Multimedia Appendix 3 Clinical profile and characteristics of health care professionals interviewed for the feasibility assessment of the Translatly digital translation platform, conducted through an ethnographic research questionnaire. This appendix provides a comprehensive statistical summary of respondents' medical specialties, clinic types, and work locations. It also details their current practices for managing language barriers in clinical settings, including the types of translation services they use and their preferred mode of access to online translation options (on-demand or appointment-based).

Multimedia Appendix 4 Anonymized data set of translation requests from the Translatly pilot phase at the University Hospital of the Goethe University in Frankfurt am Main, Germany, covering the period from December 1, 2022, to January 31, 2023. The data include the total number of translation requests (n=39), the number of completed translations (n=16), and the number of unanswered requests (n=23). The appendix also provides detailed information on the duration of the translations, the languages translated, and the medical departments from which the requests originated. This data set is fully anonymized.

## Abbreviations

AngularJS: Angular Java Script
GDPR: General Data Protection Regulation
MT: machine translation
OAuth: Open Authorization
ReactJS: React Java Script
REST API: Representational State Transfer Application Programming Interface

## Data Availability

The anonymized data supporting the findings of this study are included as Multimedia Appendix 4.
