# Supplementary material for: Digital Translation Platform (Translatly) to Overcome Communication Barriers in Clinical Care: Pilot Study
Source: JMIR Form Res. 2025 Mar 14;9:e63095. doi: 10.2196/63095 (PMC11953595; doi:10.2196/63095)
Supplement: Multimedia Appendix 1 [file formative_v9i1e63095_app1.pdf]

Multimedia Appendix 1

Ethnographic research questionnaire for medical staff

Intro [3 min]

We are currently working to develop a digital service to facilitate translation between doctors and patients. Through our questionnaire, we aim to gain a holistic view of the patient-doctor relationship and a better understanding of the general communication pathways between doctors and patients. In particular, we want to understand what currently works well and what challenges medical staff face in their clinical routine when dealing with non-native speaking patients.

Experience/Introduction [5 min]

- Tell us about yourself. Who are you? What is your role, your specialty?
- How many doctors and other medical personnel do you work with in your team/department/hospital?
- How long have you been practicing as a doctor?
- What do you love most about your field of medicine?

Patient Side: General Questions [5 min]

- Can you walk me through the process when a patient comes through your practice/hospital/department? What do patients do? Who do they interact with?
- What percentage of patients come in without an appointment compared to those who have an appointment?
- Do you use software that helps you in your work in your practice/hospital/department?
- Specifically, what do you use the software for?
- Do you use any applications on your phone while practicing? If so, which ones? And how do they help you?

Questions on Patient-Doctor Communication [10 min]

- When it comes to non-German-speaking patients, what are the most common languages that come through your clinic/practice doors?
- How would you describe the interaction and the quality of communication with a non-German-speaking patient?
- Do you know in advance (when making the appointment) that the patient does not speak German?
- If a patient does not speak German, how do you usually handle the situation?

- What do you think are the main problems in providing quality (equivalent) medical care to non-native speakers?
- How would you describe the long-term impact of this communication problem on your clinic/practice/department or your practice?

General Questions on Translation [15 min]

- If you had to choose a translator, what are the top 3 languages you would need most?
- In which cases would you need a translation service to assist you? Everyday cases? Critical cases?
- Have you ever used translation services? If so, who did you go to? Explain how it works.
- Do you remember a particular case where you wished you had translation help? If so, can you tell us about it?
- What are your ideas or experiences regarding the choice of translation assistance? External translator vs. patient's relative vs. clinic staff. What are the advantages and disadvantages of each?
- Have you used official providers (translation services, software, third parties) in your work? If so, which ones?
    - If yes, what do you think of them?
    - What worked well?
    - What didn't work well?

Translatly specific [15 min]

- Now imagine you had an app that helps translate the conversation between you and the patient in real-time, how would you imagine this app works?
- Would you use such an app? If yes, why? If no, why not?
- How often would you estimate using it, for example, in a week?
- What do you think should be the main functions of this app? And why?
- Would you prefer video or audio translation? And why?
- As a physician, what would you prefer: an immediate on-demand translation when the patient arrives? Or would you rather know in advance (when you make the appointment)? And why?
- If possible, would you try to always use the same translator for the same patient? Would you consider this option for future appointments?
- Who do you think should translate between you as a doctor and a patient? And why?

Adoption specific [10 min]

- When it comes to implementing new software/technology in your practice/hospital, what is the typical process?
- Who is usually the main contact person when it comes to deciding on new software/technology?

- How long does the purchase process for new software/technology usually take?
- Do you know of any medical regulatory issues we should be aware of when translating between doctors and patients?

Closing remarks [5 min]

Thank you for taking the time for this interview. It was very helpful to us.

- Do you have any questions for us?
- Would you like to stay in touch with us? We will keep you updated as soon as we have the first version of the app up and running. We would like you to be one of our first testers. Would you join us?
- If you know a doctor who you think would be interested in such a service, we would be very grateful if you could put us in touch with them?
