# Supplementary figures and images for: Digital Translation Platform (Translatly) to Overcome Communication Barriers in Clinical Care: Pilot Study
Source: JMIR Form Res. 2025 Mar 14;9:e63095. doi: 10.2196/63095 (PMC11953595; doi:10.2196/63095)

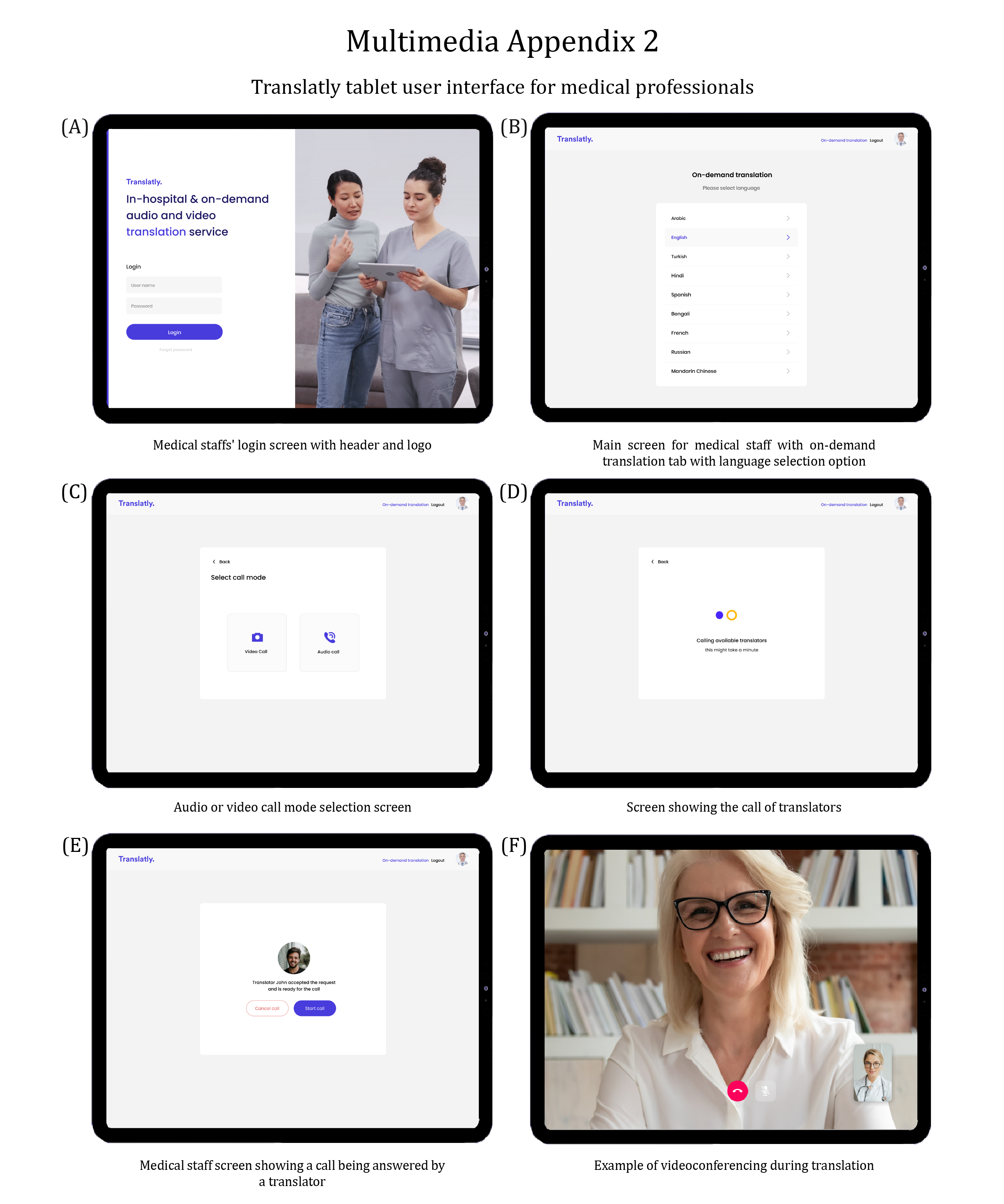

Supplement: Multimedia Appendix 2 [file formative_v9i1e63095_app2.png]
