# Supplementary material for: Digital Translation Platform (Translatly) to Overcome Communication Barriers in Clinical Care: Pilot Study
Source: JMIR Form Res. 2025 Mar 14;9:e63095. doi: 10.2196/63095 (PMC11953595; doi:10.2196/63095)
Supplement: Multimedia Appendix 3 [file formative_v9i1e63095_app3.pdf]

Multimedia Appendix 3

Ethnographic Research Questionnaire - Interviewee Characteristics (n = 10)	
**Medical Specialty**	**n (%)**
Oncologist	4 (40%)
Cardiologist	1 (10%)
Gastroenterologist	1 (10%)
Otorhinolaryngologist	1 (10%)
General internist	1 (10%)
General internist & psychotherapist	1 (10%)
Physiotherapist	1 (10%)
**Clinic Type**	**n (%)**
Maximum Care Hospital or University Clinic	6 (60%)
Private Practice	3 (30%)
Private Practice and Hospital	1 (10%)
**Location of Working Place:**	**n (%)**
Not Specified	3 (30%)
Offenbach am Main, Germany	2 (20%)
Frankfurt am Main, Germany	1 (10%)
Düsseldorf, Germany	1 (10%)
United States	1 (10%)

**Translation Services Used:**	**n (%)**
Both Medical Staff and Relatives	5 (50%)
Own Medical Staff	3 (30%)
Trained Translators Employed by the Hospital	2 (20%)
Relatives	1 (10%)
**Other Official Translation Services (Software, Third Parties) Used:**	**n (%)**
No	9 (90%)
Yes	1 (10%)
**Preferred Online Translation Option: On-demand or Appointment**	**n (%)**
On-demand	80 (80%)
Both	2 (20%)
Appointment	0 (0%)
