# Supplementary material for: Digital Translation Platform (Translatly) to Overcome Communication Barriers in Clinical Care: Pilot Study
Source: JMIR Form Res. 2025 Mar 14;9:e63095. doi: 10.2196/63095 (PMC11953595; doi:10.2196/63095)
Supplement: Multimedia Appendix 4 [file formative_v9i1e63095_app4.pdf]

Statistics for Translatly at the University Hospital of the Goethe University in Frankfurt am Main

Period 01.12.2022 – 31.01.2023:

39 translation requests

- 23 (59%) requests remained unanswered, despite being in "common" languages -> appointment feature!

- 16 (41%) completed translations:

209 (5-49) translated minutes in 16 conversations by 10 translators.

Translated languages:

1. Russian (6)
2. Ukrainian (3)
3. Yugoslav (2)
4. Arabic (2)
5. Greek (2)
6. Urdu (1)

Requests from:

1. Infectious Diseases (5)
2. Emergency Department (4)
3. Medicine 1+2 (3)
4. Pediatric Clinic (2)
5. Psychiatry (2)
